# Diaqua­(5-carb­oxy­benzene-1,3-dicarboxyl­ato-κ^2^ *O* ^1^,*O* ^1′^)(6,6′-dimethyl-2,2′-bipyridine-κ^2^ *N*,*N*′)nickel(II) hepta­hydrate

**DOI:** 10.1107/S1600536811032090

**Published:** 2011-08-17

**Authors:** Wen-Wen Shan, Han-Lin Xiong, Chong-Zhen Mei

**Affiliations:** a North China University of Water Conservancy and Electric Power, Zhengzhou 450011, People’s Republic of China

## Abstract

In the title compound, [Ni(C_9_H_4_O_6_)(C_12_H_12_N_2_)(H_2_O)_2_]·7H_2_O, the Ni^II^ atom is six-coordinated by two O atoms from a chelating carboxyl­ate group of a 5-carb­oxy­benzene-1,3-dicarboxyl­ate ligand, two O atoms of two water mol­ecules and two N atoms from a 6,6′-dimethyl-2,2′-bipyridine ligand in a distorted octa­hedral geometry. The compound exhibits a three-dimensional supra­molecular structure composed of the complex mol­ecules and lattice water mol­ecules, which are linked together by inter­molecular O—H⋯O hydrogen bonds and partly overlapping π–π inter­actions between the pyridine and benzene rings [centroid–centroid distances = 3.922 (2) and 3.921 (2) Å]. One of the lattice water mol­ecules is disordered over two positions in an occupancy ratio of 0.521 (6):0.479 (6).

## Related literature

For background to network topologies and applications of coordination polymers, see: Maspoch *et al.* (2007 ▶); Ockwig *et al.* (2005 ▶); Zang *et al.* (2006 ▶). For O—H⋯O hydrogen bonds, see: Desiraju (2004 ▶). For π–π inter­actions, see: Zang *et al.* (2010 ▶).
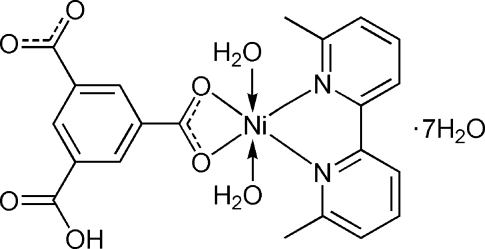


## Experimental

### 

#### Crystal data

[Ni(C_9_H_4_O_6_)(C_12_H_12_N_2_)(H_2_O)_2_]·7H_2_O
*M* *_r_* = 613.21
Monoclinic, 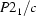

*a* = 7.4358 (5) Å
*b* = 19.9044 (7) Å
*c* = 18.7547 (12) Å
β = 100.748 (6)°
*V* = 2727.1 (3) Å^3^
*Z* = 4
Mo *K*α radiation
μ = 0.79 mm^−1^
*T* = 296 K
0.21 × 0.20 × 0.19 mm

#### Data collection

Bruker APEXII CCD diffractometer
Absorption correction: multi-scan (*SADABS*; Bruker, 2001 ▶) *T*_min_ = 0.852, *T*_max_ = 0.865
10544 measured reflections
4792 independent reflections
3924 reflections with *I* > 2σ(*I*)
*R*_int_ = 0.024

#### Refinement

*R*[*F* ^2^ > 2σ(*F* ^2^)] = 0.043
*wR*(*F* ^2^) = 0.112
*S* = 1.07
4792 reflections
359 parameters
7 restraints
H-atom parameters constrained
Δρ_max_ = 0.52 e Å^−3^
Δρ_min_ = −0.44 e Å^−3^

### 

Data collection: *APEX2* (Bruker, 2007 ▶); cell refinement: *SAINT* (Bruker, 2007 ▶); data reduction: *SAINT*; program(s) used to solve structure: *SHELXS97* (Sheldrick, 2008 ▶); program(s) used to refine structure: *SHELXL97* (Sheldrick, 2008 ▶); molecular graphics: *DIAMOND* (Brandenburg, 1999 ▶); software used to prepare material for publication: *SHELXTL* (Sheldrick, 2008 ▶).

## Supplementary Material

Crystal structure: contains datablock(s) I, global. DOI: 10.1107/S1600536811032090/hy2454sup1.cif

Structure factors: contains datablock(s) I. DOI: 10.1107/S1600536811032090/hy2454Isup2.hkl

Additional supplementary materials:  crystallographic information; 3D view; checkCIF report

## Figures and Tables

**Table 1:** Hydrogen-bond geometry (Å, °)

*D*—H⋯*A*	*D*—H	H⋯*A*	*D*⋯*A*	*D*—H⋯*A*
O3—H3⋯O6^i^	0.82	1.74	2.542 (3)	167
O1*W*—H1*WA*⋯O4^ii^	0.85	1.93	2.720 (3)	154
O1*W*—H1*WB*⋯O6*W*^iii^	0.85	1.91	2.705 (4)	156
O2*W*—H2*WA*⋯O5^iv^	0.85	2.00	2.681 (3)	136
O2*W*—H2*WC*⋯O7*W*^v^	0.85	2.02	2.729 (4)	141
O3*W*—H3*WA*⋯O5^iv^	0.85	1.99	2.827 (4)	169
O3*W*—H3*WB*⋯O9*W*^v^	0.85	2.17	2.935 (5)	150
O4*W*—H4*WA*⋯O6^iv^	1.02	1.82	2.830 (7)	170
O4*W*—H4*WB*⋯O4^vi^	0.85	2.36	3.215 (8)	179
O4*W*′—H4*WD*⋯O6^iv^	0.80	2.16	2.964 (7)	180
O4*W*′—H4*WF*⋯O6*W*^vii^	0.89	2.27	2.736 (7)	113
O5*W*—H5*WA*⋯O7*W*	0.90	2.14	2.974 (8)	155
O5*W*—H5*WC*⋯O2^iv^	0.85	2.05	2.860 (4)	159
O6*W*—H6*WC*⋯O4*W*^viii^	0.85	1.78	2.597 (8)	162
O6*W*—H6*WA*⋯O5*W*	0.75	1.94	2.687 (5)	178
O7*W*—H7*WB*⋯O8*W*	0.80	1.89	2.681 (6)	170
O7*W*—H7*WC*⋯O1*W*^ii^	0.85	2.19	2.990 (5)	158
O8*W*—H8*WB*⋯O9*W*	0.90	2.03	2.905 (6)	164
O8*W*—H8*WC*⋯O3*W*^ix^	0.85	2.31	2.916 (6)	129
O9*W*—H9*WB*⋯O1^iv^	0.85	2.14	2.968 (4)	166
O9*W*—H9*WC*⋯O3*W*^x^	0.85	2.03	2.845 (5)	162

Symmetry codes: (i) 
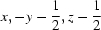
; (ii) 
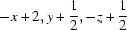
; (iii) 
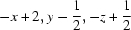
; (iv) 
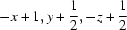
; (v) 
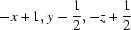
; (vi) 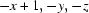; (vii) 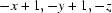; (viii) 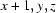; (ix) 
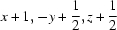
; (x) 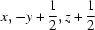.

**Fig. 1.**
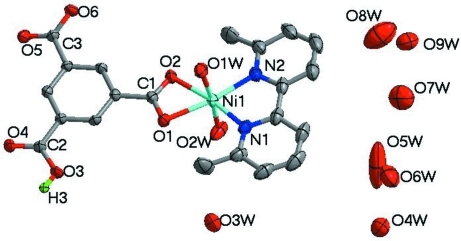
Molecular structure of the title compound. Displacement ellipsoids are drawn at the 50% probability level. Irrespective H atoms and the disordered O4W' are omitted for clarity.

**Fig. 2.**
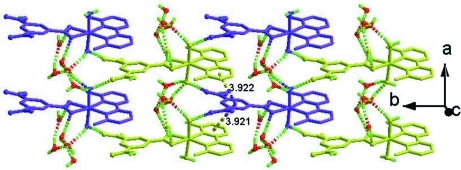
A view of the supramolecular layer in the title compound. Dotted lines represent hydrogen bonds and π–π interactions [centroid–centroid distances = 3.922 (2) and 3.921 (2) Å].

**Fig. 3.**
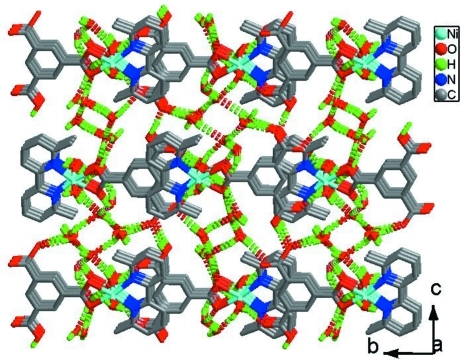
The three-dimensional supramolecular structure connected by interlayer hydrogen bonds (dotted lines).

## supplementary crystallographic information

### Comment

Metallosupramolecular chemistry has received much attention due to their variety of architectures and the potential applications as functional materials (Maspoch *et al.*, 2007; Ockwig *et al.*, 2005). The choice of ligands and metal centers can affect the final structures. A great number of organic aromatic polycarboxylate and *N*-donor ligands have been successfully employed in the generation of many novel structures (Zang *et al.*, 2006). To further explore the influence of multicarboxylate and *N*-donor ligands on the properties and construction of coordination compounds, we undertake synthetic and structural studies on the title compound, a Ni(II) complex based on 1,3,5-benzenetricarboxylic acid (H_3_btc) and 6,6'-dimethyl-2,2'-bipyridine (dmbpy).

As shown in Fig. 1, the asymmetric unit consists of one Ni^II^ atom, one Hbtc ligand, one dmbpy ligand, two coordinated and seven lattice water molecules. The Hbtc ligand occurs in a form with an intact COOH group. The Ni^II^ atom is six-coordinated by two O atoms from one chelating carboxylate group of the Hbtc ligand, two O atoms of two water molecules and two N atoms from a dmbpy ligand, completing a distorted octahedral geometry. N1, N2, O1 and O2 comprise the equatorial plane, while O1W and O2W occupy the axial positions. As depicted in Fig. 2, each complex molecule is connected to four adjacent ones through hydrogen bonds (Table 1), resulting in a two-dimensional supramolecular structure in the *ab* plane, in which partly overlapping π–π stacking interactions involving the benzene and pyridine rings are detected [centroid–centroid distances = 3.922 (2) and 3.921 (2) Å]. Adjacent layers are associated together by hydrogen bonds with the hydroxyl groups of the intact COOH groups serving as donors and the uncoordinated carboxylate O atoms from different layers as accepters. The lattice water molecules are fixed in the three-dimensional supramolecular net through hydrogen bonds (Fig. 3).

### Experimental

The title compound was synthesized hydrothermally in a Teflon-lined stainless steel container by heating a mixture of H_3_btc (0.011 g, 0.05 mmol), dmbpy (0.009 g, 0.05 mmol), Ni(NO_3_)_2_.6H_2_O (0.015 g, 0.05 mmol) and NaOH (0.004 g, 0.1 mmol) in 7 ml of distilled water at 120°C for 3 days, and then cooling it to room temperature. Green block crystals of the title compound were obtained in 75% yield based on nickel.

### Refinement

One of the lattice water molecules is disordered over two positions in a 0.521 (6):0.479 (6) ratio. H atoms of the organic ligands were positioned geometrically and refined using a riding model, with C—H = 0.93 (aromatic), 0.96 (methyl) and O—H = 0.82 Å and with *U*_iso_(H) = 1.2(1.5 for methyl and hydroxyl)*U*_eq_(C, O). H atoms of the water molecules were located from a difference Fourier map and refined as riding with *U*_iso_(H) = 1.5*U*_eq_(O).

### Crystal data

**Table:**

[Ni(C_9_H_4_O_6_)(C_12_H_12_N_2_)(H_2_O)_2_]·7H_2_O	*F*(000) = 1288
*M**_r_* = 613.21	*D*_x_ = 1.494 Mg m^−^^3^
Monoclinic, *P*2_1_/*c*	Mo *K*α radiation, λ = 0.71073 Å
Hall symbol: -P 2ybc	Cell parameters from 5756 reflections
*a* = 7.4358 (5) Å	θ = 3.1–25.1°
*b* = 19.9044 (7) Å	µ = 0.79 mm^−^^1^
*c* = 18.7547 (12) Å	*T* = 296 K
β = 100.748 (6)°	Block, green
*V* = 2727.1 (3) Å^3^	0.21 × 0.20 × 0.19 mm
*Z* = 4	

### Data collection

**Table:**

Bruker APEXII CCD diffractometer	4792 independent reflections
Radiation source: fine-focus sealed tube	3924 reflections with *I* > 2σ(*I*)
graphite	*R*_int_ = 0.024
φ and ω scans	θ_max_ = 25.0°, θ_min_ = 3.2°
Absorption correction: multi-scan (*SADABS*; Bruker, 2001)	*h* = −8→7
*T*_min_ = 0.852, *T*_max_ = 0.865	*k* = −23→23
10544 measured reflections	*l* = −22→18

### Refinement

**Table:**

Refinement on *F*^2^	Primary atom site location: structure-invariant direct methods
Least-squares matrix: full	Secondary atom site location: difference Fourier map
*R*[*F*^2^ > 2σ(*F*^2^)] = 0.043	Hydrogen site location: inferred from neighbouring sites
*wR*(*F*^2^) = 0.112	H-atom parameters constrained
*S* = 1.07	*w* = 1/[σ^2^(*F*_o_^2^) + (0.0512*P*)^2^ + 1.4022*P*] where *P* = (*F*_o_^2^ + 2*F*_c_^2^)/3
4792 reflections	(Δ/σ)_max_ < 0.001
359 parameters	Δρ_max_ = 0.52 e Å^−^^3^
7 restraints	Δρ_min_ = −0.44 e Å^−^^3^

### Fractional atomic coordinates and isotropic or equivalent isotropic displacement parameters (Å^2^)

**Table:**

	*x*	*y*	*z*	*U*_iso_*/*U*_eq_	Occ. (<1)
Ni1	0.70769 (6)	0.080884 (19)	0.25745 (2)	0.03021 (16)	
O1	0.7517 (4)	−0.01080 (10)	0.20364 (13)	0.0418 (6)	
O2	0.6738 (3)	−0.01416 (10)	0.30997 (12)	0.0346 (5)	
O3	0.7991 (4)	−0.21564 (10)	0.07083 (12)	0.0413 (6)	
H3	0.7996	−0.2377	0.0339	0.062*	
O4	0.8529 (4)	−0.31588 (11)	0.12401 (12)	0.0407 (6)	
O5	0.7135 (3)	−0.32604 (11)	0.38608 (12)	0.0403 (6)	
O6	0.7669 (4)	−0.22980 (11)	0.44653 (12)	0.0414 (6)	
O1W	0.9818 (3)	0.07458 (11)	0.30571 (13)	0.0402 (6)	
H1WA	1.0248	0.1139	0.3142	0.060*	
H1WB	0.9917	0.0530	0.3454	0.060*	
O2W	0.4405 (4)	0.07809 (12)	0.20585 (16)	0.0592 (8)	
H2WA	0.4003	0.1180	0.1991	0.089*	
H2WC	0.3779	0.0569	0.2321	0.089*	
O3W	0.2685 (6)	0.07326 (15)	0.00654 (18)	0.0889 (12)	
H3WA	0.2790	0.1069	0.0349	0.133*	
H3WB	0.2908	0.0402	0.0355	0.133*	
O4W	0.0439 (13)	0.3920 (4)	0.0141 (4)	0.0793 (16)	0.479 (6)
H4WA	0.1206	0.3496	0.0243	0.119*	0.479 (6)
H4WB	0.0705	0.3718	−0.0225	0.119*	0.479 (6)
O4W'	0.1795 (12)	0.4147 (3)	0.0175 (4)	0.0793 (16)	0.521 (6)
H4WD	0.1938	0.3757	0.0273	0.119*	0.521 (6)
H4WF	0.0831	0.4181	−0.0182	0.119*	0.521 (6)
O5W	0.6215 (7)	0.43234 (17)	0.1293 (4)	0.175 (3)	
H5WA	0.6808	0.4337	0.1758	0.262*	
H5WC	0.5221	0.4502	0.1360	0.262*	
O6W	0.9119 (5)	0.48672 (16)	0.08448 (19)	0.0840 (11)	
H6WC	0.9703	0.4542	0.0703	0.126*	
H6WA	0.8303	0.4723	0.0972	0.126*	
O7W	0.8074 (5)	0.4822 (2)	0.2739 (2)	0.0973 (12)	
H7WB	0.8414	0.4718	0.3154	0.146*	
H7WC	0.8916	0.5056	0.2610	0.146*	
O8W	0.9166 (6)	0.4315 (3)	0.4073 (3)	0.1361 (19)	
H8WB	0.8012	0.4305	0.4152	0.204*	
H8WC	0.9868	0.4048	0.4350	0.204*	
O9W	0.5399 (5)	0.45536 (17)	0.42213 (18)	0.0861 (11)	
H9WB	0.4716	0.4663	0.3823	0.129*	
H9WC	0.4442	0.4530	0.4406	0.129*	
N1	0.7626 (4)	0.16095 (13)	0.19407 (16)	0.0380 (7)	
N2	0.6634 (4)	0.15982 (13)	0.32397 (16)	0.0371 (7)	
C1	0.7209 (4)	−0.04411 (16)	0.25714 (17)	0.0301 (7)	
C2	0.8196 (4)	−0.25571 (15)	0.12681 (17)	0.0296 (7)	
C3	0.7403 (4)	−0.26418 (15)	0.38882 (17)	0.0287 (7)	
C4	0.7420 (4)	−0.11928 (15)	0.25751 (16)	0.0270 (7)	
C5	0.7759 (4)	−0.15158 (14)	0.19578 (16)	0.0274 (7)	
H5	0.7871	−0.1267	0.1549	0.033*	
C6	0.7933 (4)	−0.22150 (14)	0.19505 (16)	0.0254 (7)	
C7	0.7778 (4)	−0.25820 (15)	0.25693 (16)	0.0262 (7)	
H7	0.7881	−0.3048	0.2564	0.031*	
C8	0.7472 (4)	−0.22603 (14)	0.31937 (16)	0.0251 (7)	
C9	0.7290 (4)	−0.15617 (14)	0.31904 (16)	0.0281 (7)	
H9	0.7079	−0.1342	0.3605	0.034*	
C10	0.8084 (5)	0.15830 (19)	0.1278 (2)	0.0463 (9)	
C11	0.8383 (8)	0.0920 (2)	0.0959 (2)	0.0712 (14)	
H11A	0.9197	0.0657	0.1308	0.107*	
H11B	0.8912	0.0983	0.0535	0.107*	
H11C	0.7233	0.0690	0.0828	0.107*	
C12	0.8301 (6)	0.2173 (2)	0.0901 (2)	0.0556 (11)	
H12	0.8643	0.2151	0.0449	0.067*	
C13	0.8017 (6)	0.2778 (2)	0.1191 (3)	0.0645 (13)	
H13	0.8127	0.3171	0.0933	0.077*	
C14	0.7566 (6)	0.28089 (18)	0.1865 (3)	0.0577 (12)	
H14	0.7375	0.3222	0.2071	0.069*	
C15	0.7398 (5)	0.22160 (16)	0.2236 (2)	0.0414 (9)	
C16	0.6961 (5)	0.22106 (16)	0.2969 (2)	0.0412 (9)	
C17	0.6877 (6)	0.27930 (19)	0.3363 (3)	0.0585 (12)	
H17	0.7148	0.3207	0.3178	0.070*	
C18	0.6393 (6)	0.2752 (2)	0.4028 (3)	0.0659 (13)	
H18	0.6316	0.3140	0.4297	0.079*	
C19	0.6020 (6)	0.2140 (2)	0.4300 (2)	0.0581 (12)	
H19	0.5677	0.2112	0.4751	0.070*	
C20	0.6156 (5)	0.15593 (18)	0.3898 (2)	0.0455 (9)	
C21	0.5736 (8)	0.0888 (2)	0.4174 (2)	0.0686 (14)	
H21A	0.4809	0.0673	0.3824	0.103*	
H21B	0.5305	0.0941	0.4622	0.103*	
H21C	0.6824	0.0618	0.4254	0.103*	

### Atomic displacement parameters (Å^2^)

**Table:**

	*U*^11^	*U*^22^	*U*^33^	*U*^12^	*U*^13^	*U*^23^
Ni1	0.0398 (3)	0.0199 (2)	0.0289 (3)	−0.00149 (17)	0.00125 (18)	0.00021 (16)
O1	0.0762 (19)	0.0220 (11)	0.0287 (13)	−0.0036 (11)	0.0135 (12)	0.0041 (10)
O2	0.0500 (15)	0.0223 (11)	0.0325 (13)	0.0009 (10)	0.0104 (11)	−0.0024 (9)
O3	0.0785 (19)	0.0281 (12)	0.0199 (12)	0.0030 (12)	0.0156 (12)	−0.0016 (10)
O4	0.0666 (17)	0.0266 (12)	0.0291 (13)	0.0142 (11)	0.0092 (12)	−0.0010 (10)
O5	0.0648 (17)	0.0262 (12)	0.0289 (13)	−0.0097 (11)	0.0058 (12)	0.0044 (10)
O6	0.0755 (19)	0.0306 (12)	0.0196 (12)	−0.0032 (12)	0.0123 (12)	0.0001 (10)
O1W	0.0412 (14)	0.0314 (12)	0.0441 (15)	−0.0031 (10)	−0.0022 (11)	0.0000 (11)
O2W	0.0478 (16)	0.0412 (14)	0.078 (2)	−0.0101 (12)	−0.0174 (14)	0.0219 (14)
O3W	0.151 (4)	0.0525 (19)	0.057 (2)	0.020 (2)	0.004 (2)	−0.0123 (16)
O4W	0.105 (4)	0.068 (3)	0.065 (3)	0.021 (3)	0.013 (3)	0.006 (2)
O4W'	0.105 (4)	0.068 (3)	0.065 (3)	0.021 (3)	0.013 (3)	0.006 (2)
O5W	0.194 (5)	0.052 (2)	0.336 (8)	0.018 (3)	0.198 (6)	0.017 (3)
O6W	0.083 (3)	0.073 (2)	0.091 (3)	−0.0016 (18)	0.004 (2)	−0.0246 (19)
O7W	0.070 (2)	0.130 (3)	0.098 (3)	0.026 (2)	0.031 (2)	−0.006 (3)
O8W	0.102 (3)	0.180 (5)	0.135 (4)	0.023 (3)	0.043 (3)	0.070 (4)
O9W	0.100 (3)	0.092 (2)	0.058 (2)	0.017 (2)	−0.0040 (19)	0.0068 (19)
N1	0.0387 (17)	0.0293 (14)	0.0420 (18)	−0.0055 (12)	−0.0027 (13)	0.0060 (13)
N2	0.0342 (16)	0.0318 (15)	0.0420 (18)	0.0030 (12)	−0.0010 (13)	−0.0071 (13)
C1	0.0387 (19)	0.0241 (16)	0.0255 (17)	−0.0023 (14)	0.0006 (14)	−0.0025 (14)
C2	0.0364 (19)	0.0286 (17)	0.0243 (17)	0.0008 (14)	0.0067 (14)	0.0007 (14)
C3	0.0370 (19)	0.0264 (16)	0.0226 (17)	−0.0004 (14)	0.0054 (13)	0.0024 (13)
C4	0.0346 (18)	0.0211 (15)	0.0245 (16)	−0.0032 (13)	0.0035 (13)	−0.0002 (12)
C5	0.0392 (19)	0.0250 (15)	0.0178 (15)	−0.0002 (13)	0.0046 (13)	0.0037 (12)
C6	0.0320 (17)	0.0233 (15)	0.0201 (15)	0.0005 (13)	0.0029 (13)	0.0012 (12)
C7	0.0337 (17)	0.0207 (14)	0.0228 (16)	0.0002 (13)	0.0017 (13)	0.0010 (12)
C8	0.0320 (17)	0.0236 (15)	0.0194 (15)	−0.0022 (13)	0.0037 (13)	0.0015 (12)
C9	0.0389 (19)	0.0253 (15)	0.0208 (16)	−0.0011 (13)	0.0072 (13)	−0.0023 (13)
C10	0.048 (2)	0.047 (2)	0.041 (2)	−0.0110 (18)	−0.0007 (18)	0.0122 (18)
C11	0.117 (4)	0.060 (3)	0.042 (2)	−0.019 (3)	0.029 (3)	0.001 (2)
C12	0.050 (3)	0.057 (3)	0.056 (3)	−0.010 (2)	−0.001 (2)	0.026 (2)
C13	0.054 (3)	0.052 (3)	0.083 (4)	−0.008 (2)	−0.001 (2)	0.036 (2)
C14	0.050 (3)	0.0293 (19)	0.089 (4)	0.0000 (17)	0.002 (2)	0.015 (2)
C15	0.0311 (19)	0.0276 (18)	0.061 (3)	−0.0013 (14)	−0.0029 (17)	0.0042 (17)
C16	0.0294 (19)	0.0255 (17)	0.063 (3)	0.0015 (14)	−0.0057 (17)	−0.0059 (16)
C17	0.055 (3)	0.032 (2)	0.081 (3)	0.0043 (18)	−0.006 (2)	−0.013 (2)
C18	0.064 (3)	0.045 (2)	0.080 (4)	0.008 (2)	−0.010 (3)	−0.030 (2)
C19	0.057 (3)	0.061 (3)	0.051 (3)	0.010 (2)	−0.004 (2)	−0.027 (2)
C20	0.045 (2)	0.045 (2)	0.043 (2)	0.0077 (17)	−0.0010 (17)	−0.0136 (18)
C21	0.104 (4)	0.060 (3)	0.047 (3)	0.004 (3)	0.029 (3)	−0.008 (2)

### Geometric parameters (Å, °)

**Table:**

Ni1—O2W	2.042 (3)	N2—C16	1.360 (4)
Ni1—N2	2.070 (3)	C1—C4	1.504 (4)
Ni1—O1W	2.074 (2)	C2—C6	1.494 (4)
Ni1—N1	2.074 (3)	C3—C8	1.517 (4)
Ni1—O1	2.140 (2)	C4—C9	1.386 (4)
Ni1—O2	2.169 (2)	C4—C5	1.388 (4)
O1—C1	1.259 (4)	C5—C6	1.398 (4)
O2—C1	1.261 (4)	C5—H5	0.9300
O3—C2	1.305 (4)	C6—C7	1.394 (4)
O3—H3	0.8200	C7—C8	1.390 (4)
O4—C2	1.226 (4)	C7—H7	0.9300
O5—C3	1.247 (4)	C8—C9	1.397 (4)
O6—C3	1.265 (4)	C9—H9	0.9300
O1W—H1WA	0.8500	C10—C12	1.396 (5)
O1W—H1WB	0.8499	C10—C11	1.483 (6)
O2W—H2WA	0.8502	C11—H11A	0.9600
O2W—H2WC	0.8499	C11—H11B	0.9600
O3W—H3WA	0.8500	C11—H11C	0.9600
O3W—H3WB	0.8497	C12—C13	1.354 (6)
O4W—H4WA	1.0159	C12—H12	0.9300
O4W—H4WB	0.8499	C13—C14	1.368 (7)
O4W'—H4WD	0.8000	C13—H13	0.9300
O4W'—H4WF	0.8879	C14—C15	1.388 (5)
O5W—H5WA	0.9000	C14—H14	0.9300
O5W—H5WC	0.8500	C15—C16	1.470 (6)
O6W—H6WC	0.8499	C16—C17	1.383 (5)
O6W—H6WA	0.7500	C17—C18	1.362 (7)
O7W—H7WC	0.8500	C17—H17	0.9300
O7W—H7WB	0.8001	C18—C19	1.368 (6)
O7W—H7WC	0.8500	C18—H18	0.9300
O8W—H8WB	0.8978	C19—C20	1.395 (5)
O8W—H8WC	0.8500	C19—H19	0.9300
O9W—H9WB	0.8502	C20—C21	1.486 (6)
O9W—H9WC	0.8499	C21—H21A	0.9600
N1—C10	1.350 (5)	C21—H21B	0.9600
N1—C15	1.352 (4)	C21—H21C	0.9600
N2—C20	1.349 (5)		
			
O2W—Ni1—N2	93.23 (11)	C7—C6—C5	119.4 (3)
O2W—Ni1—O1W	174.46 (10)	C7—C6—C2	121.2 (3)
N2—Ni1—O1W	92.10 (10)	C5—C6—C2	119.3 (3)
O2W—Ni1—N1	91.57 (11)	C8—C7—C6	120.8 (3)
N2—Ni1—N1	80.36 (12)	C8—C7—H7	119.6
O1W—Ni1—N1	90.82 (10)	C6—C7—H7	119.6
O2W—Ni1—O1	88.51 (11)	C7—C8—C9	119.1 (3)
N2—Ni1—O1	170.69 (10)	C7—C8—C3	122.0 (3)
O1W—Ni1—O1	85.99 (10)	C9—C8—C3	118.9 (3)
N1—Ni1—O1	108.75 (10)	C4—C9—C8	120.6 (3)
O2W—Ni1—O2	90.35 (9)	C4—C9—H9	119.7
N2—Ni1—O2	110.07 (10)	C8—C9—H9	119.7
O1W—Ni1—O2	86.39 (9)	N1—C10—C12	120.4 (4)
N1—Ni1—O2	169.27 (10)	N1—C10—C11	119.3 (3)
O1—Ni1—O2	60.74 (9)	C12—C10—C11	120.3 (4)
C1—O1—Ni1	90.39 (19)	C10—C11—H11A	109.5
C1—O2—Ni1	89.00 (18)	C10—C11—H11B	109.5
C2—O3—H3	109.5	H11A—C11—H11B	109.5
Ni1—O1W—H1WA	109.3	C10—C11—H11C	109.5
Ni1—O1W—H1WB	109.3	H11A—C11—H11C	109.5
H1WA—O1W—H1WB	109.5	H11B—C11—H11C	109.5
Ni1—O2W—H2WA	109.2	C13—C12—C10	120.2 (4)
Ni1—O2W—H2WC	109.2	C13—C12—H12	119.9
H2WA—O2W—H2WC	109.5	C10—C12—H12	119.9
H3WA—O3W—H3WB	103.1	C12—C13—C14	119.6 (4)
H4WA—O4W—H4WB	63.5	C12—C13—H13	120.2
H4WB—O4W—H4WD	71.2	C14—C13—H13	120.2
H4WD—O4W'—H4WF	107.6	C13—C14—C15	119.1 (4)
H5WA—O5W—H5WC	97.4	C13—C14—H14	120.5
H6WC—O6W—H6WA	107.4	C15—C14—H14	120.5
H7WC—O7W—H7WB	107.6	N1—C15—C14	121.6 (4)
H7WB—O7W—H7WC	107.6	N1—C15—C16	116.3 (3)
H8WB—O8W—H8WC	112.6	C14—C15—C16	122.1 (3)
H9WB—O9W—H9WC	88.0	N2—C16—C17	121.5 (4)
C10—N1—C15	119.0 (3)	N2—C16—C15	116.2 (3)
C10—N1—Ni1	127.5 (2)	C17—C16—C15	122.3 (3)
C15—N1—Ni1	113.4 (2)	C18—C17—C16	119.0 (4)
C20—N2—C16	119.3 (3)	C18—C17—H17	120.5
C20—N2—Ni1	127.3 (2)	C16—C17—H17	120.5
C16—N2—Ni1	113.3 (2)	C17—C18—C19	120.1 (4)
O1—C1—O2	119.7 (3)	C17—C18—H18	120.0
O1—C1—C4	119.5 (3)	C19—C18—H18	120.0
O2—C1—C4	120.7 (3)	C18—C19—C20	119.7 (4)
O4—C2—O3	123.9 (3)	C18—C19—H19	120.1
O4—C2—C6	122.8 (3)	C20—C19—H19	120.1
O3—C2—C6	113.3 (3)	N2—C20—C19	120.4 (4)
O5—C3—O6	124.6 (3)	N2—C20—C21	118.6 (3)
O5—C3—C8	119.3 (3)	C19—C20—C21	121.0 (4)
O6—C3—C8	116.1 (3)	C20—C21—H21A	109.5
C9—C4—C5	120.1 (3)	C20—C21—H21B	109.5
C9—C4—C1	120.5 (3)	H21A—C21—H21B	109.5
C5—C4—C1	119.5 (3)	C20—C21—H21C	109.5
C4—C5—C6	120.1 (3)	H21A—C21—H21C	109.5
C4—C5—H5	120.0	H21B—C21—H21C	109.5
C6—C5—H5	120.0		
			
O2W—Ni1—O1—C1	−93.3 (2)	C2—C6—C7—C8	−177.8 (3)
O1W—Ni1—O1—C1	86.0 (2)	C6—C7—C8—C9	1.1 (5)
N1—Ni1—O1—C1	175.52 (19)	C6—C7—C8—C3	−176.5 (3)
O2—Ni1—O1—C1	−2.09 (18)	O5—C3—C8—C7	−19.0 (5)
O2W—Ni1—O2—C1	90.2 (2)	O6—C3—C8—C7	159.5 (3)
N2—Ni1—O2—C1	−176.26 (18)	O5—C3—C8—C9	163.4 (3)
O1W—Ni1—O2—C1	−85.34 (19)	O6—C3—C8—C9	−18.1 (4)
N1—Ni1—O2—C1	−10.2 (6)	C5—C4—C9—C8	−0.9 (5)
O1—Ni1—O2—C1	2.08 (18)	C1—C4—C9—C8	179.5 (3)
O2W—Ni1—N1—C10	−85.4 (3)	C7—C8—C9—C4	−0.3 (5)
N2—Ni1—N1—C10	−178.4 (3)	C3—C8—C9—C4	177.4 (3)
O1W—Ni1—N1—C10	89.6 (3)	C15—N1—C10—C12	−0.6 (5)
O1—Ni1—N1—C10	3.6 (3)	Ni1—N1—C10—C12	176.0 (3)
O2—Ni1—N1—C10	14.8 (7)	C15—N1—C10—C11	178.4 (4)
O2W—Ni1—N1—C15	91.4 (2)	Ni1—N1—C10—C11	−5.0 (5)
N2—Ni1—N1—C15	−1.6 (2)	N1—C10—C12—C13	−1.4 (6)
O1W—Ni1—N1—C15	−93.6 (2)	C11—C10—C12—C13	179.6 (4)
O1—Ni1—N1—C15	−179.6 (2)	C10—C12—C13—C14	1.9 (6)
O2—Ni1—N1—C15	−168.3 (5)	C12—C13—C14—C15	−0.4 (6)
O2W—Ni1—N2—C20	89.9 (3)	C10—N1—C15—C14	2.1 (5)
O1W—Ni1—N2—C20	−88.6 (3)	Ni1—N1—C15—C14	−175.0 (3)
N1—Ni1—N2—C20	−179.1 (3)	C10—N1—C15—C16	−178.0 (3)
O2—Ni1—N2—C20	−1.7 (3)	Ni1—N1—C15—C16	4.9 (4)
O2W—Ni1—N2—C16	−93.2 (2)	C13—C14—C15—N1	−1.6 (6)
O1W—Ni1—N2—C16	88.3 (2)	C13—C14—C15—C16	178.5 (4)
N1—Ni1—N2—C16	−2.2 (2)	C20—N2—C16—C17	2.3 (5)
O2—Ni1—N2—C16	175.2 (2)	Ni1—N2—C16—C17	−174.9 (3)
Ni1—O1—C1—O2	3.6 (3)	C20—N2—C16—C15	−177.5 (3)
Ni1—O1—C1—C4	−176.0 (3)	Ni1—N2—C16—C15	5.4 (4)
Ni1—O2—C1—O1	−3.6 (3)	N1—C15—C16—N2	−7.0 (5)
Ni1—O2—C1—C4	176.0 (3)	C14—C15—C16—N2	172.9 (3)
O1—C1—C4—C9	172.7 (3)	N1—C15—C16—C17	173.3 (3)
O2—C1—C4—C9	−6.9 (5)	C14—C15—C16—C17	−6.9 (6)
O1—C1—C4—C5	−6.9 (5)	N2—C16—C17—C18	−2.3 (6)
O2—C1—C4—C5	173.6 (3)	C15—C16—C17—C18	177.4 (4)
C9—C4—C5—C6	1.3 (5)	C16—C17—C18—C19	0.8 (6)
C1—C4—C5—C6	−179.1 (3)	C17—C18—C19—C20	0.6 (6)
C4—C5—C6—C7	−0.5 (5)	C16—N2—C20—C19	−0.8 (5)
C4—C5—C6—C2	176.7 (3)	Ni1—N2—C20—C19	176.0 (3)
O4—C2—C6—C7	−9.8 (5)	C16—N2—C20—C21	177.8 (4)
O3—C2—C6—C7	168.5 (3)	Ni1—N2—C20—C21	−5.5 (5)
O4—C2—C6—C5	173.0 (3)	C18—C19—C20—N2	−0.7 (6)
O3—C2—C6—C5	−8.7 (4)	C18—C19—C20—C21	−179.2 (4)
C5—C6—C7—C8	−0.6 (5)		

### Hydrogen-bond geometry (Å, °)

**Table:**

*D*—H···*A*	*D*—H	H···*A*	*D*···*A*	*D*—H···*A*
O3—H3···O6^i^	0.82	1.74	2.542 (3)	167
O1W—H1WA···O4^ii^	0.85	1.93	2.720 (3)	154
O1W—H1WB···O6W^iii^	0.85	1.91	2.705 (4)	156
O2W—H2WA···O5^iv^	0.85	2.00	2.681 (3)	136
O2W—H2WC···O7W^v^	0.85	2.02	2.729 (4)	141
O3W—H3WA···O5^iv^	0.85	1.99	2.827 (4)	169
O3W—H3WB···O9W^v^	0.85	2.17	2.935 (5)	150
O4W—H4WA···O6^iv^	1.02	1.82	2.830 (7)	170
O4W—H4WB···O4^vi^	0.85	2.36	3.215 (8)	179
O4W'—H4WD···O6^iv^	0.80	2.16	2.964 (7)	180
O4W'—H4WF···O6W^vii^	0.89	2.27	2.736 (7)	113
O5W—H5WA···O7W	0.90	2.14	2.974 (8)	155
O5W—H5WC···O2^iv^	0.85	2.05	2.860 (4)	159
O6W—H6WC···O4W^viii^	0.85	1.78	2.597 (8)	162
O6W—H6WA···O5W	0.75	1.94	2.687 (5)	178
O7W—H7WB···O8W	0.80	1.89	2.681 (6)	170
O7W—H7WC···O1W^ii^	0.85	2.19	2.990 (5)	158
O8W—H8WB···O9W	0.90	2.03	2.905 (6)	164
O8W—H8WC···O3W^ix^	0.85	2.31	2.916 (6)	129
O9W—H9WB···O1^iv^	0.85	2.14	2.968 (4)	166
O9W—H9WC···O3W^x^	0.85	2.03	2.845 (5)	162

Symmetry codes: (i) *x*, −*y*−1/2, *z*−1/2; (ii) −*x*+2, *y*+1/2, −*z*+1/2; (iii) −*x*+2, *y*−1/2, −*z*+1/2; (iv) −*x*+1, *y*+1/2, −*z*+1/2; (v) −*x*+1, *y*−1/2, −*z*+1/2; (vi) −*x*+1, −*y*, −*z*; (vii) −*x*+1, −*y*+1, −*z*; (viii) *x*+1, *y*, *z*; (ix) *x*+1, −*y*+1/2, *z*+1/2; (x) *x*, −*y*+1/2, *z*+1/2.
